# A Traumatic Ulcer Caused by Accidental Lip Biting Following Topical Anesthesia: A Case Report

**DOI:** 10.7759/cureus.38316

**Published:** 2023-04-29

**Authors:** Anushree Tiwari

**Affiliations:** 1 Dentistry, American Academy of Orthopaedic Surgeons, Rosemont, USA

**Keywords:** oral soft tissue injury, oral mucosal lesions, topical anesthesia, self-inflicting injuries, lip biting

## Abstract

Lip biting is a very common issue that dentists encounter, particularly with younger children following a dental procedure. Several studies have reported soft tissue injuries, specifically lip biting following dental treatment under local anesthesia, mostly with an inferior alveolar nerve block. However, such injury with topical anesthesia has never been reported in more than 20 years, and literature has not touched on it much. The next generation of dentists can use this case report as a reference when treating young children. Lip biting can be avoided with risk assessment and the right preventative measures; if it does occur, appropriate palliative care must be given to treat it. This case report presents a case of lip biting by a four-year-old child after undergoing a dental restorative procedure under topical anesthesia.

## Introduction

Dentists frequently see patients with lip-biting problems, especially with children. The anesthetic's effects may cause the children to bite their lips accidentally. The children may mistakenly bite their lip because they can't feel it owing to the effects of the anesthetic, or they may do it out of curiosity. Soft tissue damage, particularly lip biting after a dental procedure under local anesthetic, has been documented in a few investigations. A prospective study by College et al. stated that after a mandibular nerve block anesthesia, 13% of children between the ages of 2 and 18 developed soft tissue injuries [1]. The frequency of soft tissue damage is highest in the lowest age groups: 18% in children under the age of four, 16% in children ages four to seven, 13% in children ages 8 to 11, and 7% in children ages 12 and older [2]. However, none of the studies have reported such injury with a topical anesthetic. Parents may become alarmed by the epithelization during the healing process, as the lesion sometimes looks worrisome even though it is not. To prevent this from happening, precautions must be taken immediately after the treatment, and if it does occur, appropriate palliative care must be given to treat it.

## Case presentation

A four year-old child complained of a white, uncomfortable patch on her lower lip when she visited the dental clinic at Mission Hospital in Damoh, India. Two days ago, the patient had dental care at our department. A composite restoration was performed for the first and second molars on the lower left mandible, and the carious lesion involved enamel and superficial dentin layer. A topical anesthetic (Benzocaine 20% Oral Anesthetic Spray) was used on the buccal and lingual surfaces of the teeth. The spray tube was held 1 to 2 inches from the target, sprayed for half a second, and was allowed to stay in the area of interest for one minute before the patient spat it out. In addition, 3ml of local infiltration (2 percent lidocaine) was applied before restoration work.

**Figure 1:**
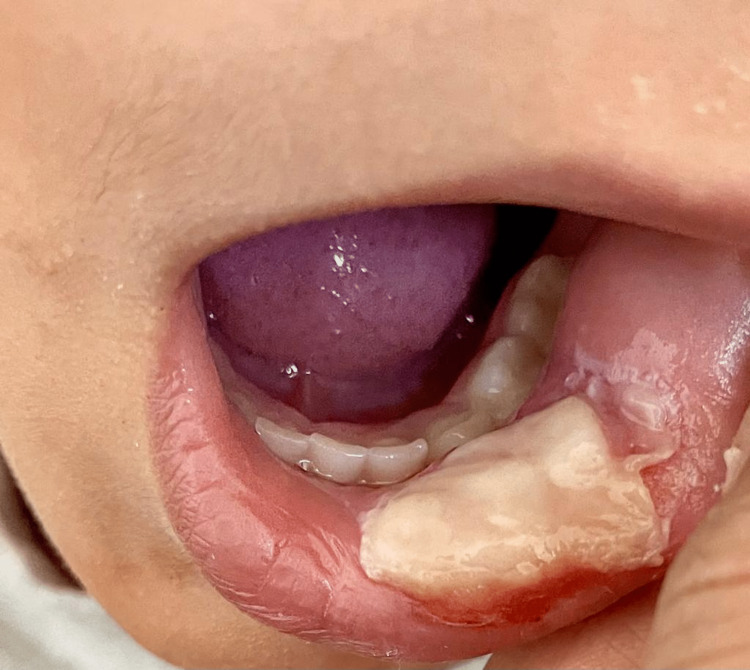
Image of the lesion on appointment day (1-2 days after biting lip).

Clinical examination after two days, when the patient returned complaining of a lesion on the lip Figure 1, revealed a white patch measuring 1 cm by 2 cm, present on the labial mucosa, extending from the midline to the corner of the mouth, trapezoidal in shape, slightly raised from the surface, and tender to the touch. The youngster was having trouble closing her lips. Differential diagnoses included herpes simplex virus infection (cold sore), traumatic fibroma, contact allergic stomatitis, and aphthous stomatitis [3]. However, since the patient was afebrile, did not have any swollen lymph nodes, did not have any history of similar lesions before, had no history of allergies or previous infections in the oral cavity, had good oral hygiene, and no history of chronic lip biting, we ruled out the lesion to be a traumatic ulcer caused by accidental lip biting following topical anesthesia.

Also, the labial mucosa and the lip cannot be numbed by local infiltration administered locally in the molar region. But the oral anesthetic spray could disperse to the lip area when spraying, and saliva is another means by which it can get there, which could have led to the numbing of the labial mucosa, thereby causing the accidental lip biting.

**Figure 2:**
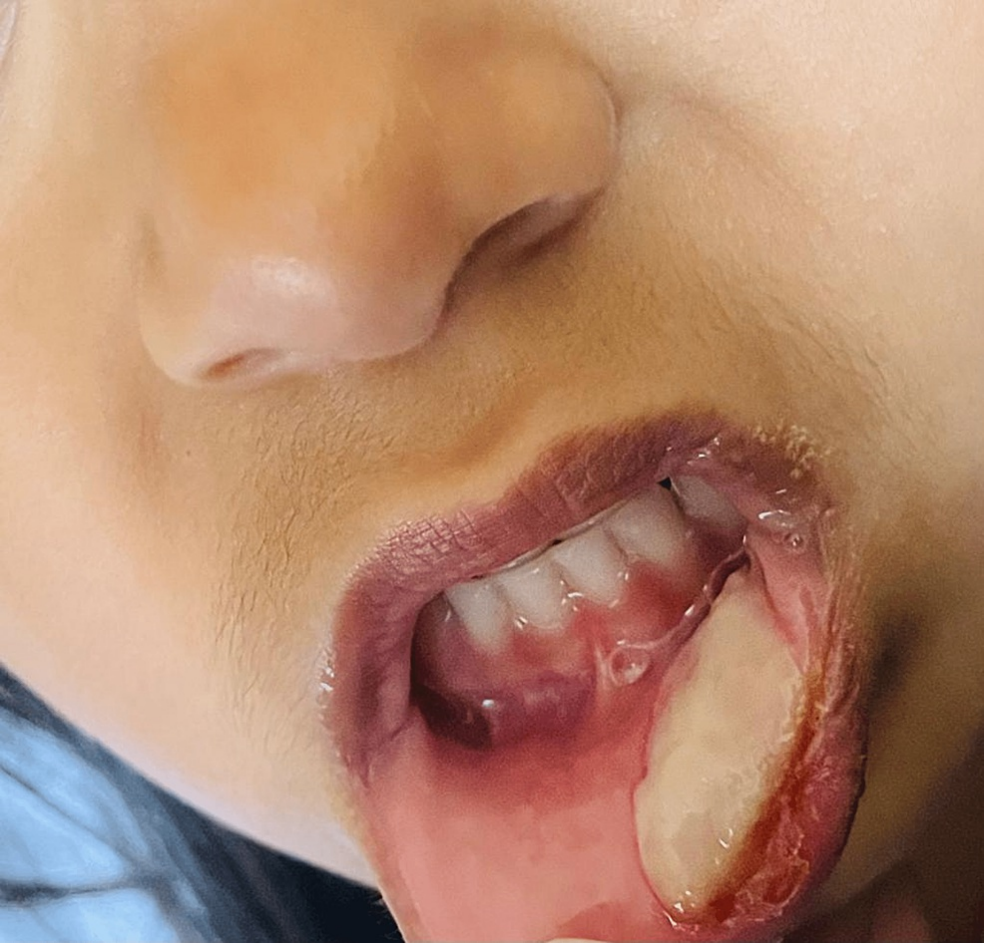
Image of the lesion in a follow-up appointment (2-3 days after biting lip).

**Figure 3:**
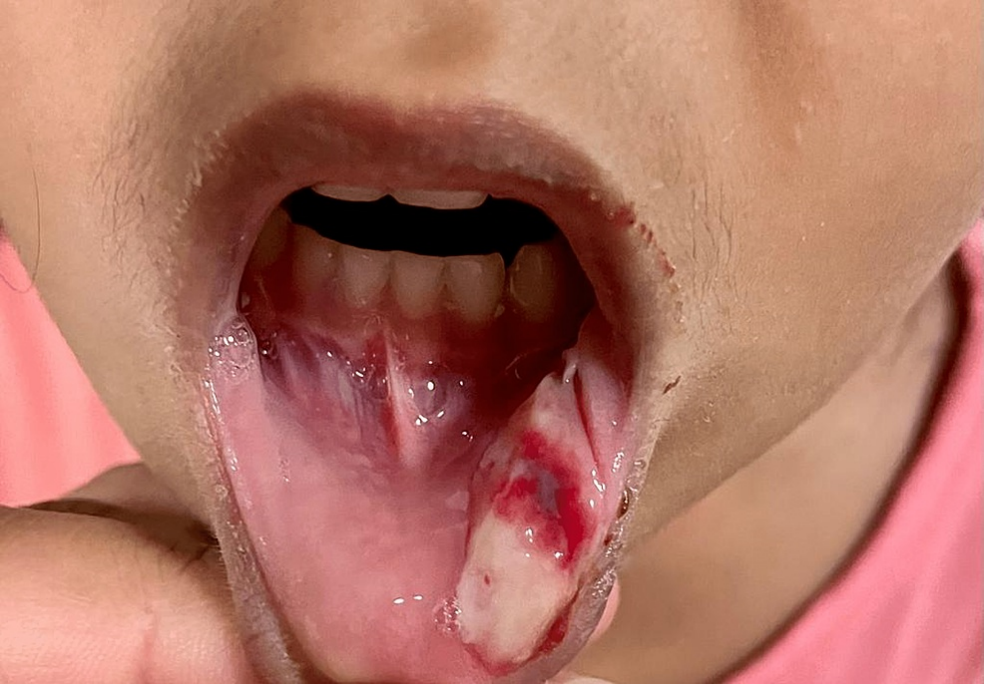
Image of the lesion in a follow-up appointment (3- 4 days after biting lip).

**Figure 4:**
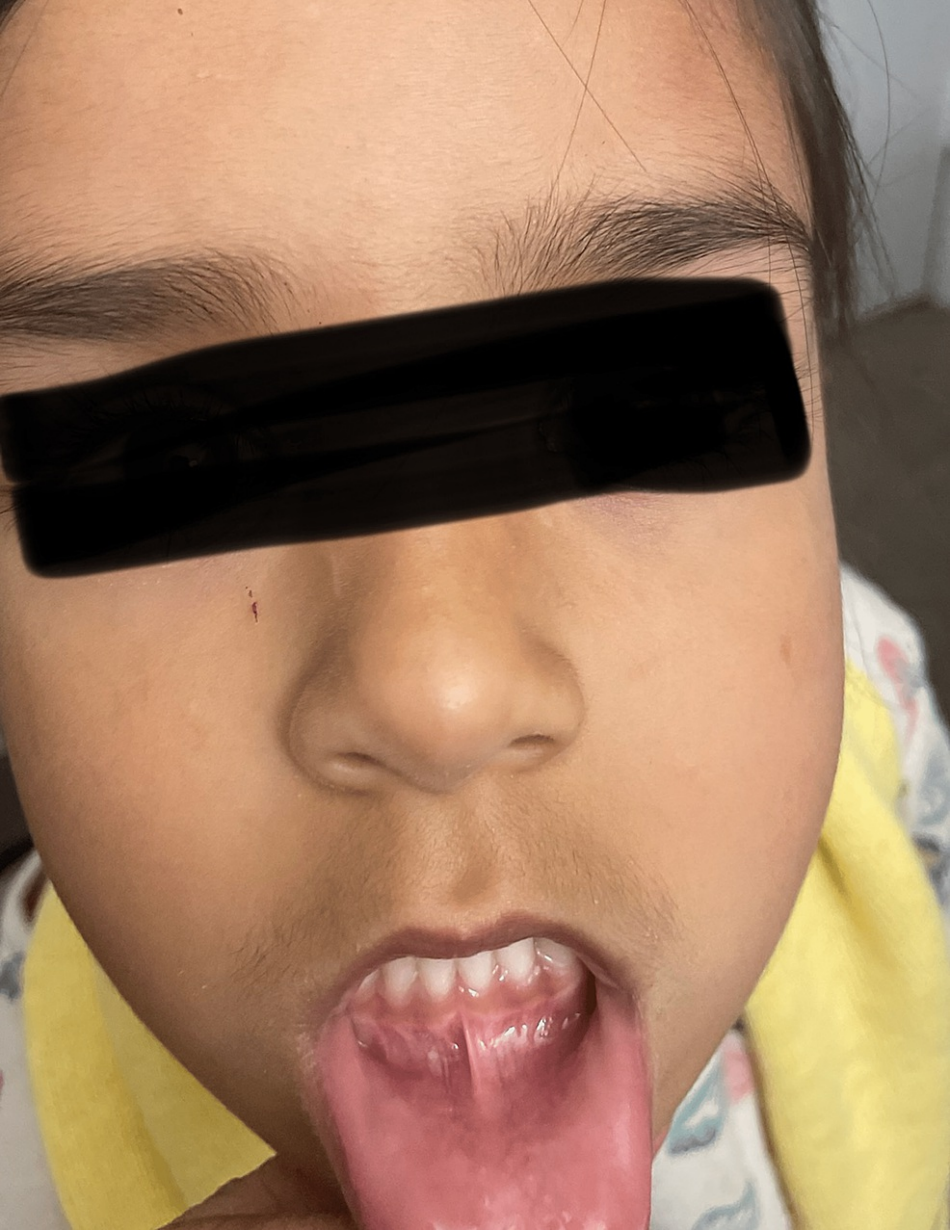
Image of the lesion in a follow-up appointment (10 days after biting lip -all healed up).

We prescribed acetaminophen oral suspension 5ml (160mg/5ml) every six hours for three days and, after that, asked to continue as needed for the patient's pain. Also, multiple daily applications of ice packs and saline rinses 2 to 3 times a day were prescribed. The patient was followed up to 12 days from the time of injury. Figure 1-4 represents the lesion's appearance on consecutive appointments. After 10 days, the lesion was fully recovered. 

## Discussion

Since children can injure themselves rather frequently, it is crucial to teach parents to keep an eye on their kids until the effects of the anesthesia have completely worn off. It should be emphasized to dentists that children should be requested to stay in the office for 1-2 hours until the numbness withers off. A cotton roll left in the labial vestibule for a while is one preventive strategy that can be employed [4]. On the other hand, if a patient with this problem shows up at your clinic after 2-4 days of the treatment, take a thorough case history. Questions regarding 'How they handled the pain and the pain and swelling during the previous two days?' etc. will aid in determining the treatment plan.

It is advised to use a palliative treatment in lip-biting situations. Palliative care can help in one to two weeks to remove the lesion. In this case, the lesion disappeared entirely within 10 days. In addition, risk assessment, prevention, and certain management strategies, as described in Table 1, can help avoid such situations [5-8].

**Table 1:** Represents the risk assessment, prevention measures, and treatment plan for lip-biting cases with topical anesthesia.

Strategy	Description
Risk Assessment:	With cognitive disorders, kids are at higher risk as they might not process the instructions. Age <4 years are too young to follow instructions. In this case, the instructions were given to the child. Even though the child was attentive and cooperative, the situation occurred.
Prevention:	Limiting the spread of topical anesthesia using cotton roles Advising the patient not to bite the lip. Instructions should be given to parents/guardians of younger children or children with cognitive disorders. Parents should be advised to keep an eye on the child till the effects of anesthesia are completely gone. Placing 2*2 cotton role in the labial vestibule after the procedure to prevent biting. Patients should be asked to wait at least an hour or two following dental treatment until the effects of anesthesia are gone.
Management:	Fabrication of lip guard to prevent lip biting, applying a clean cloth-wrapped ice cube or ice pack to the lesion [5]. This will aid in minimizing edema. Saline rinses two to three times a day [6]. Prescribe painkillers such as acetaminophen (15mg/kg per dose to be given 4-6 hourly for children) or ibuprofen (10mg/kg per dose to be given 4-6 hourly for children as needed) if there is pain. And an alcohol-free, antimicrobial mouthwash can be prescribed to keep the area clean [7]. In addition, eat soft, bland food that does not irritate the lesion, "Drink cold liquids or eat popsicles"[8]. However, if a secondary infection occurs, an antibiotic must be prescribed [7]. It should be mentioned that these lesions can worsen within 2 to 3 days of the initial onset.

## Conclusions

In some scenarios, it might become challenging when you are treating a child. Sometimes when they do not follow instructions (specifically children under 5 years of age) or sometimes when you are dealing with a special kid. However, this situation can still be avoided using an appropriate risk assessment measure and a prevention strategy. Apart from the instruction given to the parents and child themself, a dentist can do their part by adding a cotton role in the vestibule to separate the lip from teeth, in other words keeping it away from the biting areas and asking the patient to remain under observation until the effects of anesthesia are partly or completely gone.
